# Recovery through resistance? nesting urban female song sparrows (*Melospiza melodia*) have a lower glucocorticoid response to disturbance and return to parental care as quickly as rural females

**DOI:** 10.3389/fphys.2025.1520208

**Published:** 2025-04-02

**Authors:** Samuel J. Lane, Taylor E. Fossett, Isaac J. VanDiest, Kendra B. Sewall

**Affiliations:** ^1^ Virginia Tech, Department of Biological Sciences, Blacksburg, VA, United States; ^2^ Department of Biological Sciences, North Dakota State University, Fargo, ND, United States; ^3^ Virginia Tech, School of Neuroscience, Blacksburg, VA, United States

**Keywords:** glucocorticoids, corticosterone, recovery, stress resistance, parental care, urbanization

## Abstract

Urbanization represents a dramatic and relatively rapid change in the environment that has profound impacts on wild animals. Shifts in behavior and endocrine mechanisms of stress response could allow animals to successfully survive and reproduce in urban habitats. Numerous studies have examined the behavioral and physiological responses of territory-holding male songbirds to urbanization. However, breeding females likely experience anthropogenic noise, light at night, and human disturbance more frequently, and their behavioral coping responses to these disturbances are limited during incubation. Moreover, breeding females face higher energetic demands (allostatic load). Understanding how some species cope with novel urban habitats requires studying individuals facing the greatest challenges, such as breeding females. Therefore, we compared the glucocorticoid stress response and behavioral recovery from a disturbance between urban and rural female song sparrows (*Melospiza melodia*) during incubation. If facultative adjustments to the glucocorticoid stress response allow birds to cope with urban habitats, we predicted that urban females would return to parental care behaviors after a standardized stressor as soon or sooner than rural females, and that urban females would have a lower glucocorticoid response to the stressor. We captured female song sparrows at the end of the incubation period and measured their glucocorticoid (corticosterone) levels at baseline and after 30 min of standardized restraint. Concurrently, we installed radio frequency identification (RFID) systems at the nest to capture the time to return to parental care behaviors. We found that incubating urban females had significantly lower corticosterone levels when controlling for sampling timepoint (baseline and restraint-induced) compared to rural. Nest return times did not differ across habitats, and latency to return was not significantly correlated with corticosterone levels. Our findings are consistent with prior work in breeding male song sparrows at our study sites; urban males provide higher parental care and have lower restraint-induced corticosterone levels. The absence of a relationship between glucocorticoids and behavior makes it unlikely that these hormones directly regulate parental care, but lower corticosterone levels in urban birds could reflect stress resistance, which has been hypothesized to permit animals to breed in challenging or novel conditions such as urban habitats.

## 1 Introduction

As urban expansion continues, acclimating to novel urban habitats characterized by unpredictable stressors is imperative to the survival of many species (Seto et al., 2012; Lowry et al., 2013; Bonier, 2023). Urbanization is predicted to expand rapidly and have profound impacts on songbirds (Marzluff, 2001; Isaksson, 2018; Seto et al., 2012; Bonier, 2023). Though individuals of some species face compromised fitness in urban areas (De Satgé et al., 2019; Muller et al., 2020; White et al., 2022), many species cope through behavioral adjustments such as avoidance of novel stressors (Sih et al., 2011; Sol et al., 2013; Sol et al., 2018). In addition to behavioral adjustments, facultative changes in physiological mechanisms such as the glucocorticoid stress response can facilitate response to and recovery from stressors, including disturbances of greater frequency, intensity, or novelty in modified habitats (Sinclair et al., 2022).

Glucocorticoids are metabolic hormones maintained continuously at basal levels by the hypothalamic-pituitary-adrenal axis to support an individual’s energetic needs (Siegel, 1980; Sapolsky et al., 2000; Kuo et al., 2015; McEwen, 2001 Romero et al., 2009). In response to acute or chronic stressors glucocorticoids increase significantly, activating a host of behavioral and physiological mechanisms to recover homeostasis (Siegel, 1980; Sapolsky et al., 2000; Romero et al., 2009). Elevated baseline levels of glucocorticoids relative to the population can indicate chronic stress while peak levels are associated with the magnitude of an individual’s response to acute, potentially life-threatening stressors (Sapolsky et al., 2000; Small et al., 2017). Though glucocorticoids facilitate recovery from stressors, chronically elevated levels can be damaging if they shift an individual into allostasis or an ‘emergence life history stage’ (Wingfield et al., 1998; McEwen and Wingfield, 2003; Wingfield, 2005; Romero et al., 2009). In extreme cases, chronic high levels of glucocorticoids can impair cognition and behavior, even becoming neurotoxic (Sapolsky, 2015).

Individuals vary in their glucocorticoid levels, stress recovery, and potential risk from the negative effects of glucocorticoids (Sapolsky et al., 2000; Lin et al., 2004; McEwen, 2001; Bonier et al., 2009; Dickens et al., 2009; Lynn et al., 2010; Vitousek et al., 2019). The risk of damage from chronically elevated glucocorticoids can be mitigated through stress resilience or stress resistance, both of which should allow animals to maintain normal behaviors, such as breeding, despite experiencing stressors (Wingfield and Sapolsky, 2003; DuRant et al., 2013). Stress resilience is the physiological capacity to experience stress and maintain normal behaviors and is associated with faster clearance of glucocorticoids and return to basal physiological and behavioral states (Taff et al., 2018; Taft et al., 2019; Vitousek et al., 2019; Zimmer et al., 2019). Stress resistance, in contrast, is a reduction in the glucocorticoid response, either through psychological (perceptual or attentional) processes or reduced sensitivity in the glucocorticoid cascade resulting in lower hormone release (Siegel, 1995; Wingfield et al., 1995; Wingfield and Sapolsky, 2003; Wingfield et al., 2011). Either stress resilience or resistance could reduce exposure to glucocorticoids when stressors are intense and/or frequent, such as in urban areas, and could help animals breed successfully in the face of novel challenges.

Urban habitats contain stimuli that, when replicated in captive laboratory studies, elicit a glucocorticoid stress response in songbirds. Chronic anthropogenic noise, increased light at night, frequent human disturbance, and exposure to domestic predators (e.g., house cats) all increase glucocorticoids relative to baseline or controls under experimental conditions (Saunders et al., 1991; Sinclair et al., 2002; Chace and Walsh, 2006; McKinney, 2006; Loss et al., 2013; Lowry et al., 2013; Renthlei et al., 2017; Rosenberg et al., 2019; Dammhahn et al., 2020). Comparisons of the glucocorticoid stress response in male songbirds living in urban and rural areas have sought to identify glucocorticoid response patterns that could explain their ability to cope with urban habitats. However, no clear patterns have emerged across species (Bonier, 2012; Injaian et al., 2020; Sinclair et al., 2022; Bonier, 2023). Some species show higher glucocorticoids in urban areas, consistent with chronic stress in these novel habitats (Bonier, 2012; Injaian et al., 2020). It is possible that some species cope with stressors in urban habitats with greater stress resilience, which can be reflected in faster clearance of glucocorticoids in response to a synthetic glucocorticoid (i.e., dexamethasone challenge). This hypothesis has not been well-tested, but we found no evidence for faster hormone clearance in urban male song sparrows (*Melospiza melodia*) and are unaware of other empirical studies of stress resilience in urban birds (Lane et al., 2021). In contrast, stress resistance is the suppression of glucocorticoids despite external stressors and could protect against interruptions to reproduction and the potential harm from chronically elevated glucocorticoids (Siegel, 1995; Wingfield et al., 1995; Wingfield and Sapolsky, 2003; Wingfield et al., 2011). Though few studies have pursued this alternative hypothesis, some have reported or predicted lower glucocorticoids in urban male songbirds, which could be consistent with stress resistance (Bonier, 2012; Injaian et al., 2020; Sinclair et al., 2022; Grunst et al., 2014).

If stress resistance underpins the capacity for some species to persist in urban habitats, then birds in urban areas should have lower baseline and stress (restraint) induced glucocorticoids compared to rural birds. Additionally, they should recover normal behavior as quickly, if not faster, after disturbance. Importantly, this should be true even for individuals facing higher allostatic load. In contrast to male songbirds, most breeding female songbirds in the North Temperate Zone decrease their mobility while incubating eggs and brooding offspring, which should increase their exposure to disturbances because, unlike males, they cannot avoid stressors through behavioral coping mechanisms. This should be especially true for females in urban areas where disruptions are more frequent and intense (Marzluff, 2001; Baudains and Lloyd, 2007; Seress and Liker, 2015; Isaksson, 2018). Breeding females also face higher physical demands from reproduction, and sufficiently high glucocorticoid levels can induce nest abandonment or suppress reproduction completely, reducing females’ fitness (Silverin, 1986; Spée et al., 2011; Wingfield and Silverin, 1986; Love et al., 2004; Ouyang et al., 2012). It can be adaptive to suppress a stress response that might interfere with reproduction in some circumstances such as the relatively short breeding seasons of the Temperate Zone (Holberton and Wingfield, 2003; Romero, 2002; Wingfield and Sapolsky, 2003; Wada et al., 2006). Exploring how the glucocorticoid stress response and behavioral recovery from a disturbance differ among urban and rural living female birds during incubation will provide insight into the scope of facultative variation of the glucocorticoid stress response and the role of the glucocorticoid pathway in helping animals cope with rapid anthropogenic environmental change.

To explore the potential for stress resistance to underpin urban acclimation in some songbird species, we compared the glucocorticoid stress response and latency to recover from a stressor in breeding female song sparrows from replicate urban and rural study sites. Prior studies of male song sparrows show that urban males at our sites have lower corticosterone (the primary avian glucocorticoid) after a standardized stressor than rural males during the breeding season (Lane et al., 2021). Further, breeding urban and rural male song sparrows at our sites do not show differences in stress resilience measured in a dexamethasone challenge assay (Lane et al., 2021). Thus, our prior studies in male song sparrows are consistent with the hypothesis that urban song sparrows have greater stress resistance and do not elevate glucocorticoids as much as rural males in response to stressors. A critical test of this hypothesis is to measure variation in stress physiology in individuals under higher allostatic load and a greater risk of chronic glucocorticoid exposure and interference with reproductive efforts, such as breeding females (Wingfield, 2005; Goymann and Wingfield, 2004; Hämäläinen et al., 2015; Sterling, 2012; Edes et al., 2018). Therefore, in the current study, we exposed incubating female song sparrows to a controlled stressor (capture and restraint) and measured their circulating corticosterone levels at baseline and after 30 min, as well as their latency to return to the nest. Female song sparrows select a social partner at the start of the breeding season and raise 1-3 clutches, though they do seek extra-pair mating’s (Nice, 1937; Nice, 1943; O’Connor et al., 2006). Though males assist with feeding young, females do all the incubation, which requires them to remain at the nest nearly continuously from lay through hatch. Song sparrow egg incubation is ∼12–14 days long, and nestlings take approximately 10 days to fledge (Nice, 1937; Nice, 1943). With time for nest building, each breeding attempt last ∼30 days. We predicted that urban females would have lower corticosterone at baseline and in response to a controlled stressor, consistent with stress resistance. Importantly, if differences in the glucocorticoid stress response are adaptive, they should preserve normal behavior, namely, reproductive effort. Thus, we predicted that urban females would return to the nest as soon or sooner than rural females after being disrupted. However, facultative changes to the stress response system can be energetically costly, so we expected that urban females would have poorer body condition (indicative of higher allostatic load and compromised self-maintenance), compared to rural female song sparrows. Studying the stress physiology of female songbirds breeding in urban habitats is a critical step toward a holistic understanding of the consequences of urbanization for wild birds.

## 2 Methods

### 2.1 Nest searching, subject capture, and sampling

To examine the relationship between female corticosterone levels and behavioral recovery times, we located active nests during the 2020 and 2021 breeding seasons (March–July). Starting in March of each year, we searched 6 previously established field sites (3 urban and 3 rural) in Southwestern Virginia, USA that vary along an urban-rural gradient (Davies and Sewall, 2016; Davies et al., 2018). We used methods described in Seress et al. (2014) to characterize the levels of urbanization at our sites. Using principal component analysis that considered factors such as the percent of impermeable surfaces, vegetation, and building density, we created an urbanization index for each site (see Davies et al. (2018) for more detailed information on these calculations for our sites; VanDiest et al. (2024) for sizes of our field sites and density of song sparrows). Our sites range from being adjacent to one another in the case of two urban sites, to up to ∼14 km apart and song sparrows are generally at higher density in our rural sites (see Lane et al. (2024) for a map of our field sites). Nests were located through behavioral observations and systematic searching (Martin and Geupel, 1993; Lane et al., 2024). Once nests were located and a nest stage was established, we waited until late incubation (Incubation is approximately 12–14 days, (Nice, 1937; Nice, 1943), and we waited until day 10–15 of incubation; Krause et al., 2015) to catch adult females. On the day of capture, two mist nets were set up near the nest location before dawn, and the female was flushed from the nest into a mist net after sunrise. If the female was not on the nest during the initial approach, the area was observed until she was located, and a second flushing attempt was conducted after she returned to the nest. We did not attempt to flush a female more than twice in 1 day, and always waited at least 2 days between attempts. In total we caught 23 urban and 14 rural, and all birds were only sampled once.

Within 5 min of capture, we successfully collected a baseline blood sample *via* brachial venipuncture with a 26-gauge needle, and collected with heparinized capillary tubes from 21 urban and 12 rural females. Following this, we affixed a passive integrative transponder (PIT) tag to the tarsometatarsus of each bird following methods developed by Bridge and Bonter (Bridge and Bonter, 2011; Bridge et al., 2019). We then took morphometric measures (mass, head length, bill width, bill length, tarsus length, wing chord, and tail length) and restrained the female in a cloth bag for a total of 30 min from the time of capture (which has been shown to induce maximal release of corticosterone; Wingfield et al., 1992; Romero and Romero, 2002) while we installed a radio frequency identification (RFID) system at the focal female’s nest (Lane et al., 2021; Lane et al., 2024). At the end of the 30 min of restraint, we took a second brachial blood sample and released the female. We left the area immediately and did not return for 48 h, at which time we collected the data from the RFID system. Of the captured females, 20 urban and 9 rural returned to the nest following capture, (though RFID failed at 2 urban nests, resulting in 18 return times). In total, 5 urban and 4 rural nests were subsequently abandoned, though there was some overlap between groups and outcomes. We used the time from flushing the female from the nest until her return was registered by the RFID system as the latency to recover parental behavior. We monitored nest outcomes (i.e., predation, or nest destruction in urban habitats) following the protocols established in Lane et al., 2024.

### 2.2 Corticosterone assay

We quantified total baseline and stress-induced corticosterone levels using two commercially available enzyme-linked immunosorbent assay (Lot No: 04122104A, Enzo Life Sciences, Inc., Farmingdale, NY) following the manufacturer’s instructions. We previously validated the assay for song sparrows (Davies and Sewall, 2016; Davies et al., 2018). Briefly, we diluted samples 1:40 and added, 1% steroid displacement reagent (used to free the steroid from carrier proteins). We assayed samples in duplicate, with all samples from a given bird assigned to the same plate and all birds randomly assigned to one of 2 plates, with pooled standards run in duplicate on both plates. Intra-assay variation was 10.82%, inter-assay variation was 7.18%, and assay sensitivity was 27 pg/mL.

### 2.3 Statistical analysis

We conducted statistical analyses using R (R Core Team 2023, v. 4.3.1). We analyzed the effect of habitat on baseline and stress-induced levels of corticosterone using linear mixed-effects models (LMM) and linear models (LM). We fitted all LMM’s using the package “lme4” (Bates et al., 2015) and examined the residuals from each model for normality. We tested the significance of fixed effects from the LMMs using the lmerTest (Kuznetsova et al., 2017) package, which estimates degrees of freedom (df) with the Satterthwaite approximation. Model assumptions were examined using the “check_model” function within the “performance” package (Lüdecke et al., 2021). Full summaries for all models are included in the supplemental materials. For each model, backwards stepwise variable selection was used to limit the number of variables to those with a p-value of 0.2 or lower (Wang et al., 2007). All final models are presented in the supplemental materials.

#### 2.3.1 Habitat-based effects on maternal hormone levels

To determine if urban and rural female song sparrows in the incubation phase of reproduction differed in glucocorticoid levels at baseline or in response to standard restraint stress, we compared the overall relationship between corticosterone and habitat type using a linear mixed effects model (Model 1). We also ran a linear model (LM) to compare the absolute change in corticosterone between habitat types (Model 2). In this analysis and throughout, corticosterone levels, and change in corticosterone was examined in sperate models because it expressed high levels of collinearity. We normalized corticosterone levels using a natural log transformation because residuals from the initial analysis violated the assumptions of normality. In each model _ln_cort, or _ln_absolute change in cort was used as the response variable. In the initial model examining baseline and stress-induced corticosterone as a repeated measure, sample (baseline or stress-induced) and habitat type were included as fixed effects, and the birds’ band number was specified as the random effect. In this initial model, there was an interaction indicated between sampling timepoint (baseline or stress-induced) and habitat type, though it was not significant, so we dropped the interaction and moved forward with the additive model. We also included year, day of year, the time of day the bird was captured, and the time between capture and first blood sample collection in seconds (hereafter initial bleed latency) as fixed-effect covariates in both models. Year and day of year were included to control for seasonal effects of corticosterone levels. Time of day was included to control for circadian changes in corticosterone, and initial bleed latency to control for differences in baseline levels of corticosterone caused by variation in the time between capture and sample collection. However, following variable selection, day of year, capture time, and initial bleed latency were removed from both models, and year was removed from model 2.

#### 2.3.2 Behavioral recovery in response to acute stress across habitat types

To determine if urban and rural females differed in behavioral recovery from a disturbance and if/how behavioral recovery related to corticosterone levels, we fitted one generalized linear model (GLM) with a Gamma distribution, as the residuals from the initial model with a gaussian distribution were not normal. Recovery time (min.) was used as the response variable. We analyzed the effect of habitat type, baseline, and stress-induced corticosterone (Model 3) on behavioral recovery following a standard acute stressor (capture and restraint). In this model, and models where both baseline and stress-induced cort were predictors, there were moderate levels of collinearity between these variables. Day of year, and year, were initially included as fixed-effect covariates, but were removed following variable selection. Next, we explored the effect of habitat type and adult corticosterone levels on the probability of nest abandonment. We used three GLM’s fitted to a binomial distribution. Nest abandonment (0 or 1) was the response variable, with habitat type (Model 4), baseline and stress-induced corticosterone (Model 5), and body condition (Model 6) as the main effect in each model. Day of year and year were included as fixed effect covariates in each model, except for the model examining maternal body condition, where day of year was excluded because day of year was a significant predictor of body condition (Supplementary Figure S1).

#### 2.3.3 Behavioral and physiological correlates of maternal body condition across habitat types

We explored how female body condition (measured as scaled mass index (Peig and Green, 2009)) related to nest return rates, habitat type, maternal corticosterone levels, and nest abandonment. Following methods in Peig and Green, 2009, we examined Pearson correlations between body mass (g) and more fixed body measures (i.e., head length, bill width, bill length, tarsus length, wing chord, and tail length). We determined that, of the available variables, head length was the best predictor of body mass (*R* ^2^ = 0.42) and was used that as the secondary variable in the calculation of scaled mass index. We then ran one linear model (Model 7), with scaled mass index as the response variable, and habitat type, nest return rates, baseline, and stress-induced corticosterone as the predictor variables. Day of year and year were included as fixed effect covariates in the initial model, but year was removed following variable selection.

## 3 Results

### 3.1 Habitat-based effects on maternal hormone levels

**FIGURE 1:**
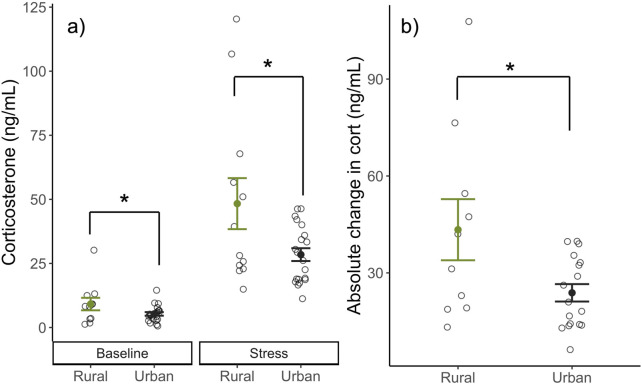
Corticosterone levels (ng/mL) of nesting birds at baseline, and stress-induced levels **(A)** and the absolute change in corticosterone (ng/mL) from baseline to stress-induced **(B)** between urban (black) and rural (green) habitats. Urban birds had lower overall corticosterone levels when controlling for sampling time point (baseline or stress-induced) and absolute change in corticosterone when compared to rural. Means ±1 standard error are shown with individual data points indicated by open circles.

We found that urban females had lower corticosterone levels overall compared to rural females (β_Habitat:Urban_ = −0.48 ± 0.20, *t*_33.15_ = −2.41, p = 0.02; Figure 1A) when controlling for sampling time point (baseline or stress-induced levels). Further, urban females had significantly less change in corticosterone from baseline to after restraint (i.e., lower stress reactivity) compared to rural (β_Habitat:Urban_ = −0.51 ± 0.23, *t*_25_ = −2.20, p = 0.037; Figure 1B).

### 3.2 Behavioral recovery in response to acute stress across habitat types

**FIGURE 2:**
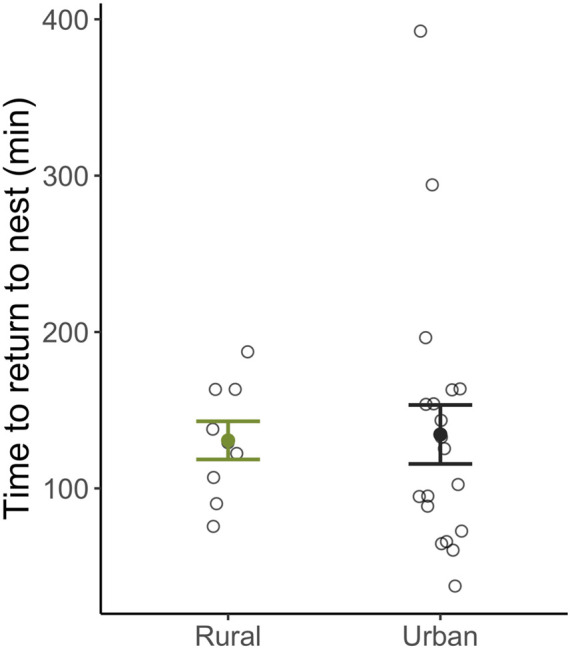
The relationship between latency to return after a stressor and habitat type. Urban and rural female expressed similar returns times following a standardized stressor. Means ±1 standard error are shown with individual data points indicated by open circles.

**FIGURE 3:**
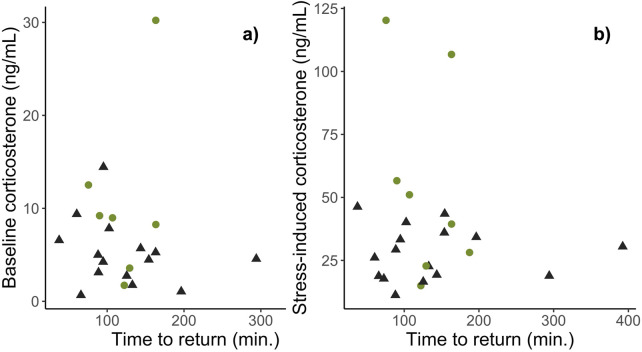
The association between maternal nest return times (min.) following acute stress and baseline **(A)** and stress-induced **(B)** levels of corticosterone, between urban (black) and rural (green) habitats. There was no association between behavioral recovery times and corticosterone levels in urban or rural dwelling female song sparrows.

The time it took females to return to maternal care behaviors following capture and restraint did not differ between habitats (β_Habitat: Urban_ = 4.70e^−4^ ± 2.18e^−3^, *t*_19_ = 0.22, p = 0.83; Figure 2) nor was it related to baseline (β_Habitat: Urban_ = 1.69e^−4^ ± 2.43e^−4^, *t*_19_ = −0.70, p = 0.50; Figure 3A) or stress-induced levels of corticosterone (β_Habitat: Urban_ = 5.30e^−5^ ± 6.35e^−5^, *t*_19_ = 0.84, p = 0.42; Figure 3B). Importantly, nest abandonment was not related to habitat type (β_Habitat: Urban_ = −0.11 ± 1.20, *z*_34_ = −0.10, p = 0.92), or corticosterone levels (Baseline: β_cort_ = 0.89 ± 0.67, *z*_24_ = 1.33, p = 0.19; Stress: β_cort_ = −0.31 ± 0.21, *z*_24_ = −1.49, p = 0.14). Rather, nest abandonment was negatively associated with day of year (β_Day of year_ = −0.23 ± 0.09, *z* = −2.61, p = 0.01) such that females nesting earlier in the season were significantly more likely to abandon their nest following capture.

### 3.3 Behavioral and physiological correlates of maternal body condition across habitat types

**FIGURE 4:**
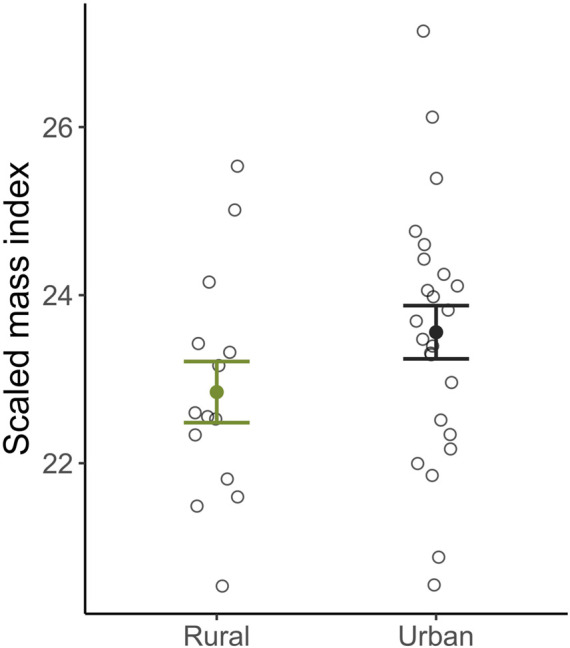
Scaled mass index body condition of urban (black) and rural (green) female song sparrows at the end of incubation. There was no difference in this measure of body condition between females residing in urban or rural habitats. Means ±1 standard error are shown with individual data points indicated by closed circles.

Female body condition at the end of incubation did not differ between habitats (β_Habitat: Urban_ = 0.28 ± 0.85, *t*_13_ = 0.34, p = 0.74; Figure 4), or correlate with return times (β_time to return_ = −0.01 ± 0.01, *t*_13_ = −1.08, p = 0.30), nor did it correlate with corticosterone at baseline (β_Baseline_ = −0.02 ± 0.09, *t*_13_ = −0.20, p = 0.84) or after a standard stressor (β_Baseline_ = −0.001 ± 0.02, *t*_13_ = −0.07, p = 0.95). Interestingly, nest abandonment was negatively correlated with maternal body condition (β_SMI_ = −1.62 ± 0.66, *z*_35_ = −2.50, p = 0.01), females with worse body conditions were more likely to abandon their nest following a standardized stressor. However, maternal body condition was positively correlated with day of year (*R* ^2^ = 0.40, p = 0.01) and day of year was also a strong predictor of nest abandonment, making it unclear if day of year or body condition is the best predictor of the likelihood to return to maternal effort.

## 4 Discussion

The implicit assumption that urban habitats are more “stressful” is logical, but neither hormonal nor fitness data unanimously uphold this inference (Injaian et al., 2020; Kurucz et al., 2021; Bonier, 2023; Deviche et al., 2023; Iglesias-Carrasco et al., 2020). Songbirds in urban habitats do not universally have higher glucocorticoids at baseline or in response to a stressor, nor do they have poorer body condition or compromised fitness (Liker et al., 2008; Bókony et al., 2012; Foltz et al., 2015; Meillère et al., 2015; Goodchild et al., 2022). However, most work in songbirds has focused on males, who face lower reproductive costs in the North Temperate Zone and can avoid urban stressors with behavioral coping responses not available to incubating or brooding females. Any negative effects created by the disturbances and challenges inherent to urban habitats should be most evident in the individuals with the greatest allostatic load, such as breeding female songbirds. Therefore, we compared the glucocorticoid stress response, latency to return to parental behavior, and body condition at the end of the incubation period between urban and rural female song sparrows. We hypothesized that urban females would have greater stress resistance (a dampened glucocorticoid stress response) to minimize exposure to glucocorticoids, which can suppress female reproduction, induce nest abandonment, or cause other pathologies (Sapolsky et al., 2000; Wingfield and Sapolsky, 2003; Lin et al., 2004; McEwen, 2001; Bonier et al., 2009; Dickens et al., 2009; Lynn et al., 2010).

We found evidence that the glucocorticoid stress response system differs between urban and rural female song sparrows. Urban female song sparrows had lower corticosterone levels when controlling for sampling timepoint (baseline and after standardized restraint), resulting in a lower overall change in corticosterone in response to a stressor, consistent with the hypothesis that urban female song sparrows show stress resistance (Figures 1A, B). Few studies have examined the corticosterone levels of breeding females residing in urban and rural habitats. Outside the breeding season, Partecke et al. found that urban and rural female European blackbirds (*Turdus merula*) had similar baseline levels of corticosterone, but that urban females had significantly lower stress-induced levels of corticosterone compared to rural females (Partecke et al., 2006). Similarly, Atwell et al. found that urban dwelling female juncos (*Junco hyemalis*) had similar baseline levels of corticosterone, but lower stress-induced corticosterone compared to rural females (Atwell et al., 2012). Conversely, Heppner et al. found that female house wrens (*Troglodytes aedon*) in urban sites have elevated baseline corticosterone during brooding compared to females at rural sites (Heppner et al., 2023). Finally, Bonier et al. found that baseline corticosterone in female, white-crowned sparrows (*Zonotrichia leucophrys*) breeding in urban areas did not differ from rural females, though breeding urban males did have higher levels than rural males (Bonier et al., 2007). Though there is no single pattern of facultative changes in the glucocorticoid system associated with urbanization, our work and similar research demonstrates that breeding urban female songbirds may rely upon facultative adjustments to the glucocorticoid pathway.

This is also evident in studies that isolate specific urban stressors (i.e., artificial light at night, ambient noise) to see their effect on free living breeding female songbirds. Several field experiments have found evidence of physiological suppression of glucocorticoids in response to chronic urban stressors. Injaian et al., 2018 found that female tree swallow (*Tachycineta bicolor*) exposed to chronic, elevated traffic noise subsequently released less corticosterone in response to acute stress. Similarly, Kleist et al., 2018 found that traffic noise was associated with lower baseline corticosterone levels of breeding females in three species (Ash-throated flycatcher, *Myiarchus cinerascens*; Western bluebird, *Sialia mexicana*; Mountain bluebird, *Sialia currucoides*), though it was associated with higher stress-induced corticosterone in the nestlings. Other studies have reported increases in glucocorticoids in response to experimental stressors. Artificial light at night was associated with elevated baseline corticosterone in great tits (*Parus major*; Ouyang et al., 2015). Additionally, Strasser and Heath, 2013 found that human disturbance increased baseline cort levels in breeding female American Kestrels (*Falco sparverius*). Future comparative studies of urban breeding songbirds should examine patterns of corticosterone, stress resilience, and resistance as a function of the intensity and recency of urbanization. This would allow us to understand if different strategies emerge when there is more time or less cost to making facultative physiological adjustments (Cockrem, 2013; Sinclair et al., 2022). Additionally, comparative approaches could reveal how life history influences patterns of glucocorticoid response because stress resilience and resistance are predicted when breeding opportunities are limited, and the relative payoff of a given breeding attempt is high (Crespi et al., 2013; Crossin et al., 2016).

Perhaps not surprisingly, given their low corticosterone levels, urban females in our study returned to incubate their nests just as quickly as rural females after restraint stress (Figure 2). Thus, female parental behavior was maintained in urban habitats, consistent with facultative differences in the glucocorticoid stress response being adaptive. However, corticosterone levels were not correlated with latency to return to the nest (Figures 3A, B), indicating that this is not the only mechanism regulating behavior. Further, and counter to our predictions, urban females did not have compromised body condition at the end of incubation (Figure 4), nor were corticosterone levels related to female body condition. Additionally, though future work should rule out stress resilience in female song sparrows using a dexamethasone challenge, our current findings are consistent with stress resistance in urban female song sparrows, a mechanism that has been proposed to protect females from the potential harm of chronically elevated glucocorticoids and is argued to ensure reproductive effort (Wingfield and Sapolsky, 2003). A dampened stress response could be adaptive in urban environments, where human presence/disturbance is high, yet predation rates are significantly lower compared to rural habitats. In our system, we found that our urban sites have significantly lower nest predation rates, but significantly higher human disturbance/presence when compared to rural sites (Lane et al., 2024; Foltz et al., 2015). Theory suggests that stress resistance might allow a female to continue incubating and brooding and benefit from rearing young in an area with lower nest predation, despite higher rates of human disturbance. Our current study attempted to measure behavioral recovery in females using return rates but future studies could build off of this by quantifying how much disturbance a female will endure on a nest before leaving (e.g., flight initiation distance) and the relationship among brooding behavior, corticosterone levels, habitat type, and nest success.

We found no differences in nest abandonment rate between habitats. Importantly, nest abandonment was not associated with corticosterone levels or magnitude of change in corticosterone but was predicted by day of the year. Females nesting earlier in the year were significantly more likely to abandon their nest than females nesting later in the season, regardless of habitat. However, females had lower body condition earlier in the season (Supplementary Figure S1), and body condition was associated with the likelihood of nest abandonment (i.e., females with lower body condition were more likely to abandon their nest) making it unclear if abandonment was an effect of season or condition. Our breeding populations are year-round residents, so migration should not affect their body condition, but nest success can change with day of year (Grant et al., 2005; Grimm et al., 2015; Grudinskaya et al., 2022), presumably because environmental conditions (e.g., temperatures, rainfall, higher food abundance) change over the season (VanDiest et al., 2024). As song sparrows often have multiple broods, females nesting earlier in the season under harsher conditions could in theory go on to nest again later in the season, where conditions could be more favorable. Thus, we cannot determine if abandonment is caused by body condition or if female body condition is simply correlated with ecological conditions over the breeding season (Breuner and Hahn, 2003). Moreover, prior work has found evidence for female body condition being both positively (Bleeker et al., 2005) and negatively (Ouyang et al., 2012) correlated with abandonment, suggesting females may abandon in some cases to conserve resources for future breeding opportunities, and in other cases because they do not have the physiological capacity to continue and attempt.

If female songbirds of some species, such as song sparrows, respond to urban habitats with stress resistance, it could be mediated by several possible underlying mechanisms. Stress resistance can be psychological in nature: reduced attention to stimuli, dampened peripheral sensation, or altered central perception can mediate habituation to novel conditions (Sapolsky et al., 2000; Herman, 2013). Essentially, if stimuli are not detected or are not perceived as stressors, they cannot activate the stress response (Sapolsky et al., 2000; Wingfield and Sapolsky, 2003). Stress resistance can also be mediated by physiological processes: alterations to the hypothalamic-pituitary-adrenal (HPA) axis can block this cascade and reduce the secretion of glucocorticoids (Sapolsky et al., 2000; Wingfield and Sapolsky, 2003; Hong et al., 2011). Such HPA axis suppression can occur if a signal from the periphery is received by the hypothalamus or septum, but the signal fails to amplify because it is not transduced to the pituitary or adrenals. This suppression can result from depleted or lowered production and release of hormones, or increased clearance of signaling molecules, including Corticotropin Releasing Hormone, Adrenocorticotropic hormone, or glucocorticoids themselves (Hauger and Dautzenberg, 2000; Wingfield and Sapolsky, 2003; Lattin et al., 2016; Vuppaladhadiam et al., 2020). As the glucocorticoid stress cascade interacts with targets such as arginine vasotocin and neuropeptide Y, changes in other systems regulating behavioral and physiological coping could also be modulated (Kuenzel et al., 2020; Heilig, 2004; Hirsch and Zukowska, 2012). Alternatively, reduced expression of receptors for hormones can decrease sensitivity in the HPA axis cascade and attenuate the glucocorticoid and associated responses (Saldanha and Silver, 1998; Spencer et al., 2009; Lattin et al., 2016; Rensel and Schlinger, 2020; Watts, 2020). Future studies could measure signaling molecules and their receptors in the brain and adrenals to examine the alterations to the HPA axis and integrated physiological cascades as it relates to urbanization (Deviche et al., 2023). Though we have no relevant data in female song sparrows, our prior work showed that urban adult male song sparrows had lower relative glucocorticoid receptor mRNA expression in the hippocampus than rural males, which suggests altered receptor expression could contribute to changes in this cascade (Lane et al., 2021). Our present finding of lower corticosterone at baseline and in response to a standardized stressor in urban females is consistent with adaptive stress resistance that protects their breeding effort. However, studies of the cellular and molecular regulation of the HPA axis are needed to fully test this hypothesis.

## 5 Conclusion

The present study provides evidence of adaptive stress resistance in breeding urban female song sparrows. Though we outline several future avenues of testing, our current findings are consistent with the hypothesis that a facultative shift in the glucocorticoid response preserves reproductive effort in altered habitats. Females in urban sites had lower overall glucocorticoid levels when controlling for sampling timepoint (baseline and stress-induced corticosterone), and returned to the nest just as quickly and maintained their reproductive effort. Surprisingly, this facultative shift in the glucocorticoid stress response did not come at a measurable cost to body condition. Though the best conclusion given these data is that urban song sparrows show stress resistance, several studies are needed to confirm this hypothesis. First, the alternative hypothesis of stress resilience needs to be tested in female urban and rural song sparrows to determine whether this competing hypothesis can be refuted. Second, mechanisms of psychological stress resistance need to be tested. Third, mechanistic evidence of stress resistance such as reduced HPA axis signaling, or decreased receptor density would support the conclusion of stress resistance in urban song sparrows. Finally, the greatest impact of this study may be that other researchers will consider stress resistance as a possible adaptive response to anthropogenic environmental change. Future comparative studies across systems could resolve how the intensity or recency of urbanization may predict whether the glucocorticoid system shifts toward greater resilience or resistance. Additionally, life history traits such as how diet, breeding strategies, and migration may predict how changes in the glucocorticoid response align with environmental change across different species. However, developing these theoretical perspectives requires many empirical studies across diverse study systems.

## Data Availability

The raw data supporting the conclusions of this article will be made available by the authors, without undue reservation.
