# Supplementary material for: Recovery through resistance? nesting urban female song sparrows (Melospiza melodia) have a lower glucocorticoid response to disturbance and return to parental care as quickly as rural females
Source: Front Physiol. 2025 Apr 2;16:1520208. doi: 10.3389/fphys.2025.1520208 (PMC11999856; doi:10.3389/fphys.2025.1520208)
Supplement: Supplementary file 1 [file Supplementaryfile1.docx]

Supplementary Material

# Supplementary Figures and Tables

# 1.1. Supplementary Tables

| ***Habitat-based effects on maternal hormone levels.*** | | | | | |
| --- | --- | --- | --- | --- | --- |
|  |  |  |  |  |  |
| Table S1: Full model Corticosterone across habitat types | | | | | |
| Response Variable: ln(Corticosterone (ng/mL)) | | |  |  |  |
| Random effect | Variance | SD |  |  |  |
| Bande Number | 0.19 | 0.44 |  |  |  |
| Residual | 0.21 | 0.46 |  |  |  |
| Number of obs: 60 | Groups: Band Number, 34 | | |  |  |
|  |  |  |  |  |  |
| Fixed Effects | Estimate | SE | Df | t value | P-value |
| Intercept | 2.13 | 0.21 | 40.88 | 10.19 | 0.03 |
| Sample: Stress | 1.84 | 0.12 | 29.22 | 15.42 | **<0.001** |
| Habitat Type: Urban | -0.48 | 0.20 | 33.15 | -2.41 | **0.02** |
| Year:2021 | -0.47 | 0.19 | 33.60 | -2.46 | **0.02** |
|  |  |  |  |  |  |
| Table S2: Change in corticosterone across habitat types. | | | |  |  |
| Response Variable: ln(Absolute change in corticosterone (ng/mL)) | | | | |  |
|  |  |  |  |  |  |
| Fixed Effects | Estimate | SE | Df | t value | P-value |
| Intercept | 3.57 | 0.19 | 19.25 | 0.00 | 0.30 |
| Habitat Type: Urban | -0.51 | 0.23 | -2.20 | 0.04 | **0.03** |

|  |  |  |  |  |  |
| --- | --- | --- | --- | --- | --- |
| ***Behavioral recovery in response to acute stress across habitat types*** | | | | | |
|  |  |  |  |  |  |
| Table S3: Nest return times between habitats | | |  |  |  |
| Response Variable: Time to return to nest (min). | | |  |  |  |
|  |  |  |  |  |  |
| Fixed Effects | Estimate | SE | Df | t value | P-value |
| Intercept | 0.007 | 0.003 | 19.00 | 2.68 | 0.02 |
| Stress-induced cort (ng/mL) | 0.00005 | 0.00006 | 19.00 | 0.83 | 0.42 |
| Baseline cort (ng/mL) | -0.0002 | 0.00024 | 19.00 | -0.69 | 0.50 |
| Habitat Type: Urban | 0.0005 | 0.002 | 19.00 | 0.22 | 0.83 |
|  |  |  |  |  |  |
| Tabl S4 Nest abandonment and habitat type | | |  |  |  |
| Response Variable: Probability of nest abandonment (0/1) | | | |  |  |
|  |  |  |  |  |  |
| Fixed Effects | Estimate | SE | Df | z value | P-value |
| Intercept | 30.45 | 11.54 | 37.00 | 2.64 | 0.01 |
| Habitat Type: Urban | 0.11 | 1.20 | 37.00 | 0.10 | 0.92 |
| Julian Day-of-year | -0.23 | 0.09 | 37.00 | -2.61 | **0.01** |
| Year:2021 | -3.46 | 1.59 | 37.00 | -2.18 | **0.03** |
|  |  |  |  |  |  |
| Table S5: Nest abandonment and corticosterone levels | | | |  |  |
| Response Variable: Probability of nest abandonment (0/1) | | | |  |  |
|  |  |  |  |  |  |
| Fixed Effects | Estimate | SE | Df | z value | P-value |
| Intercept | 67.73 | 47.17 | 24.00 | 1.44 | 0.15 |
| Julian Day-of-year | -0.47 | 0.34 | 24.00 | -1.37 | 0.17 |
| Year:2021 | -9.39 | 6.32 | 24.00 | -1.49 | 0.14 |
| Stress-induced cort (ng/mL) | -0.31 | 0.21 | 24.00 | -1.49 | 0.14 |
| Baseline cort (ng/mL) | 0.89 | 0.67 | 24.00 | 1.33 | 0.18 |

|  |  |  |  |  |  |
| --- | --- | --- | --- | --- | --- |
| ***Behavioral and physiological correlates of maternal body condition across habitat types*** | | | | | |
|  |  |  |  |  |  |
| Table S6: Body condition across habitat types | | |  |  |  |
| Response Variable: Body condition (SMI) | | |  |  |  |
|  |  |  |  |  |  |
| Fixed Effects | Estimate | SE | Df | t value | P-value |
| Intercept | 19.12 | 3.33 | 13.00 | 5.74 | 0.00 |
| Baseline cort (ng/mL) | -0.02 | 0.09 | 13.00 | -0.20 | 0.84 |
| Habitat Type: Urban | 0.28 | 0.850 | 13.00 | 0.34 | 0.74 |
| Stress-induced cort (ng/mL) | 0.04 | 0.02 | 13.00 | 1.60 | 0.13 |
| Julian Day-of-year | 0.001 | 0.02 | 13.00 | 0.07 | 0.95 |
| Time to Return (min) | -0.007 | 0.007 | 13.00 | -1.07 | 0.30 |
|  |  |  |  |  |  |
| Table S7: Does body condition predict nest abandonment? | | | |  |  |
| Response Variable: Probability of nest abandonment (0/1) | | | |  |  |
|  |  |  |  |  |  |
| Fixed Effects | Estimate | SE | Df | z value | P-value |
| Intercept | 36.58 | 15.05 | 33.00 | 2.43 | 0.02 |
| Scale Mass Index | -1.62 | 0.66 | 33.00 | -2.47 | **0.01** |
| Year:2021 | -1.78 | 1.18 | 33.00 | -1.51 | 0.13 |

## Supplementary Figures


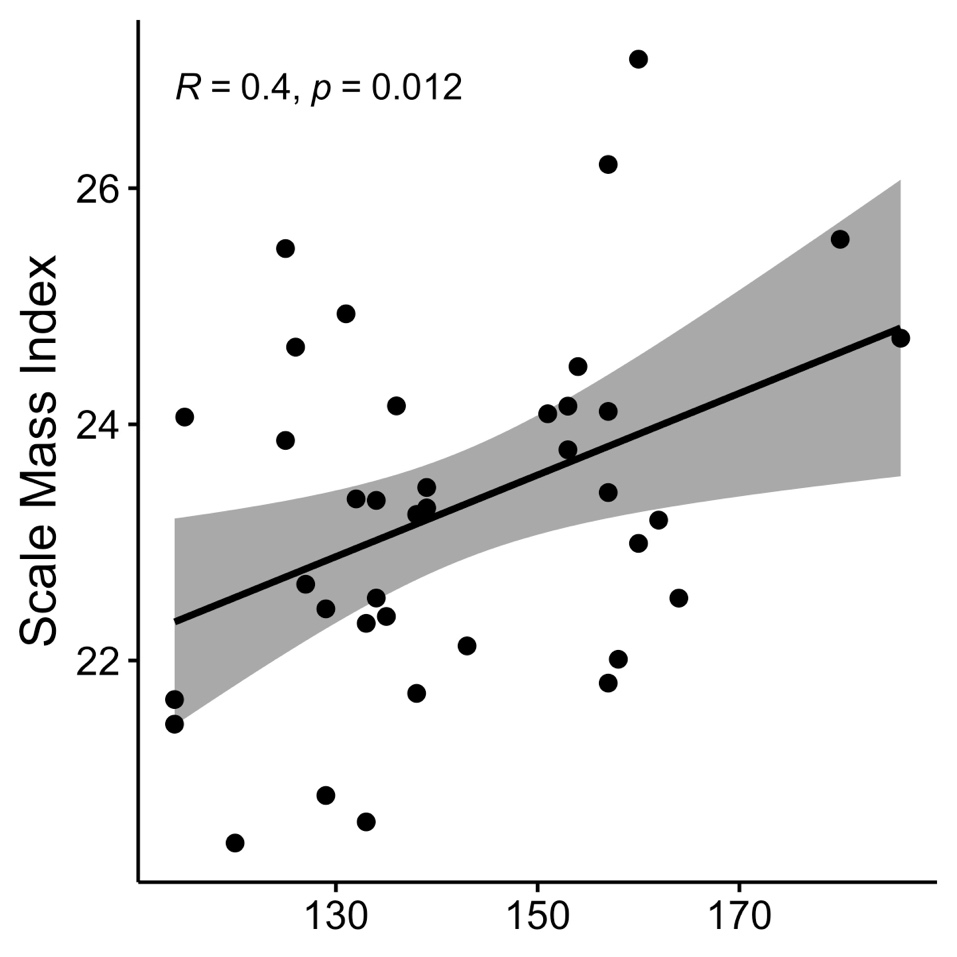


**Supplementary Figure 1.** The relationship between female body condition and day of year. Female body condition was positively associated with day of year across the breeding season.
